# Presidential Symposium: Interdisciplinary Perspectives From Early-Career Scholars on Why Age Matters

**DOI:** 10.1093/geroni/igaa057.3041

**Published:** 2020-12-16

**Authors:** Darina Petrovsky, Danielle Waldron, Katie Sakel

## Abstract

The 2020 ESPO Presidential Symposium features interdisciplinary perspectives and recent scientific advances made by early career researchers from each of the GSA scientific sections. They provide examples of how their work is addressing ways age matters. The first paper by Justice (Biological Sciences) will present the latest geroscience research. The second presentation will explore how age influences end-of-life care (Health Sciences, Starr). The third presentation will focus on age as a predictor of kidney function decline (Behavioral and Social Sciences, Surachman). The fourth presentation will explore how age shapes older adults’ resilience (Academy for Gerontology in Higher Education, Bouchard). The fifth presentation will examine opioid use in older adults (Social Research, Policy, and Practice, Jansen). Additionally, early career scholars will share information about their research trajectories and future directions within their disciplines. Given the diverse nature of these presentations, attendees will be exposed to varying strategies, methodologies, and tools that are employed across disciplines. The symposium concludes with a discussion on ways to identify synergies across different fields and promote strategies for successful cross-disciplinary collaboration.

